# Feasibility, safety, and reliability of exercise testing using the combined arm-leg (Cruiser) ergometer in subjects with a lower limb amputation

**DOI:** 10.1371/journal.pone.0202264

**Published:** 2018-08-13

**Authors:** Elisabeth K. Simmelink, Johan B. Wempe, Jan H. B. Geertzen, Lucas H. V. van der Woude, Rienk Dekker

**Affiliations:** 1 University of Groningen, University Medical Center Groningen, Department of Rehabilitation Medicine, Groningen, The Netherlands; 2 University of Groningen, University Medical Center Groningen, Department of Pulmonary Diseases, Groningen, The Netherlands; 3 University of Groningen, University Medical Center Groningen, Center for Human Movement Sciences, Groningen, The Netherlands; University of Illinois at Urbana-Champaign, UNITED STATES

## Abstract

**Background:**

Physical fitness of patients with a lower limb amputation predicts their walking ability and may be improved by physical exercise and training. A maximal exercise test is recommended prior to training in order to determine cardiovascular risks and design exercise programs. A potentially suitable ergometer for maximal exercise testing in patients with a lower limb amputation is the combined arm-leg (Cruiser) ergometer. The aim of this study was to determine feasibility, safety, and reliability of (sub)maximal exercise testing on the Cruiser ergometer in subjects with a lower limb amputation.

**Methods and findings:**

Subjects with a lower limb amputation performed 1 submaximal exercise test and 3 maximal exercise tests on the Cruiser ergometer. Feasibility was determined by examining whether key variables such as power output, heart rate and oxygen uptake were correctly and reliably measured, by determining whether a test was a maximal aerobic performance, by studying reasons for non-completion, and by measuring gross efficiency. Safety was analyzed by recording complications, electrocardiogram results, and blood pressure. Reliability was tested by comparing the results of the second and third maximal exercise test. Seventeen subjects (14 men and 3 women) out of 21 preselected subjects completed the study. In general, the maximal Cruiser exercise test was feasible. Almost 75% of the subjects reached a maximal aerobic performance. The test was also safe because no complications occurred, although electrocardiogram and blood pressure could only be reliably recorded in most subjects just before and after the test. Reliability was good: Intraclass correlation was 0.84 for peak oxygen uptake.

**Conclusions:**

The Cruiser ergometer is a feasible, safe, and reliable ergometer for measuring physical fitness of subjects with a lower limb amputation.

## Introduction

Patients who require a lower limb amputation (LLA) are often elderly and have a high prevalence of comorbidities, especially cardiovascular diseases [1]. The presence of cardiovascular diseases reduces the chance of being able to walk with a prosthesis and negatively influences mobility outcomes after LLA [2]. Other factors that influence the ability to walk with a prosthesis are amputation level, age and physical fitness [3]. Most patients with a LLA experience a decline in physical fitness, which in turn negatively influences their functional activity level [4,5,6]. In addition, energy costs of walking with a prosthesis are much higher compared with normal walking and increase proportionally with the level of amputation [4]. It has been demonstrated that maximal aerobic capacity, which is a major constituent of physical fitness, is an important predictor for walking ability in patients with a LLA due to vascular disease [5]. Furthermore, exercise training can improve walking ability [6,7].

Before starting exercise training, a maximal exercise test is not only recommended [8] for reasons of safety, especially with regard to cardiovascular risks, but also for developing individually tailored exercise programs. To achieve the best possible outcomes, the cardiovascular system has to be maximally stressed by using the largest possible muscle mass, i.e., by obtaining the highest possible oxygen uptake (peak VO_2_) and/or work capacity [9]. Patients with a LLA have lower functional muscle mass by definition. Previous studies have researched the use of different ergometers by LLA patients, including the arm ergometer [10] and the unilateral bicycle ergometer [5,7]. A disadvantage of the arm ergometer is that only the arms and part of the upper body are used. Similar to the arm ergometer, the bicycle ergometer also only involves the muscle mass of one extremity (the leg) and part of the upper body. Patients with a LLA often need help to make the cycling movement with one leg and have difficulty maintaining balance. A combined arm-leg ergometer, the Cruiser ergometer, is a suitable alternative for testing the physical fitness of patients with a unilateral LLA [11]. The Cruiser ergometer has several advantages for its users: they are seated on the ergometer and their back and residual limb are supported; they can exercise without the help of a therapist; and they use the muscle mass of the trunk and 3 extremities, i.e., 1 leg and 2 arms. The use of relatively high muscle mass during exercise on a combined arm-leg ergometer may lead to a higher peak VO_2_ [12–14]. Previous research has demonstrated that the Cruiser ergometer is a valid, reliable, and safe instrument for measuring the physical fitness of healthy volunteers [12]. The next step is to study the Cruiser in subjects with a LLA. Therefore, the aims of the current study are 1) to explore the feasibility of the Cruiser ergometer in maximal exercise testing in subjects with a LLA, 2) to evaluate the safety of the Cruiser ergometer, and 3) to study the test-retest reliability of a repeated maximal exercise test on the Cruiser ergometer.

## Materials and methods

### Population

Twenty-one subjects with a LLA (18 men and 3 women) living in the North of the Netherlands were screened for participation this study. The principal investigator (ES) informed specialists in Rehabilitation Medicine and certified prosthetists working in the North of the Netherlands about this study. These specialists and prosthetists subsequently asked subjects with a LLA to participate in the study and provided them with written information. Following the subjects’ agreement to participate, the principal investigator (ES) contacted the subjects to screen for inclusion and exclusion criteria. Inclusion criteria were: age between 18 and 75 years and a LLA (unilateral transfemoral amputation, knee disarticulation, or transtibial amputation). Exclusion criteria were: coronary heart disease, clinically relevant arrhythmia, hypertension (diastolic blood pressure > 100 mm Hg or systolic blood pressure >180 mm Hg), recently diagnosed pulmonary embolism, bilateral LLA, upper limb amputation, and cognitive impairments leading to inability to cooperate or inability to obtain consent [8].

Prior to their participation, subjects signed an informed consent form. All tests were conducted in accordance with the Declaration of Helsinki. The local Medical Ethics Committee (METc UMC Groningen) approved the study (file number METc 2011/123).

### Instruments

**Fig 1:**
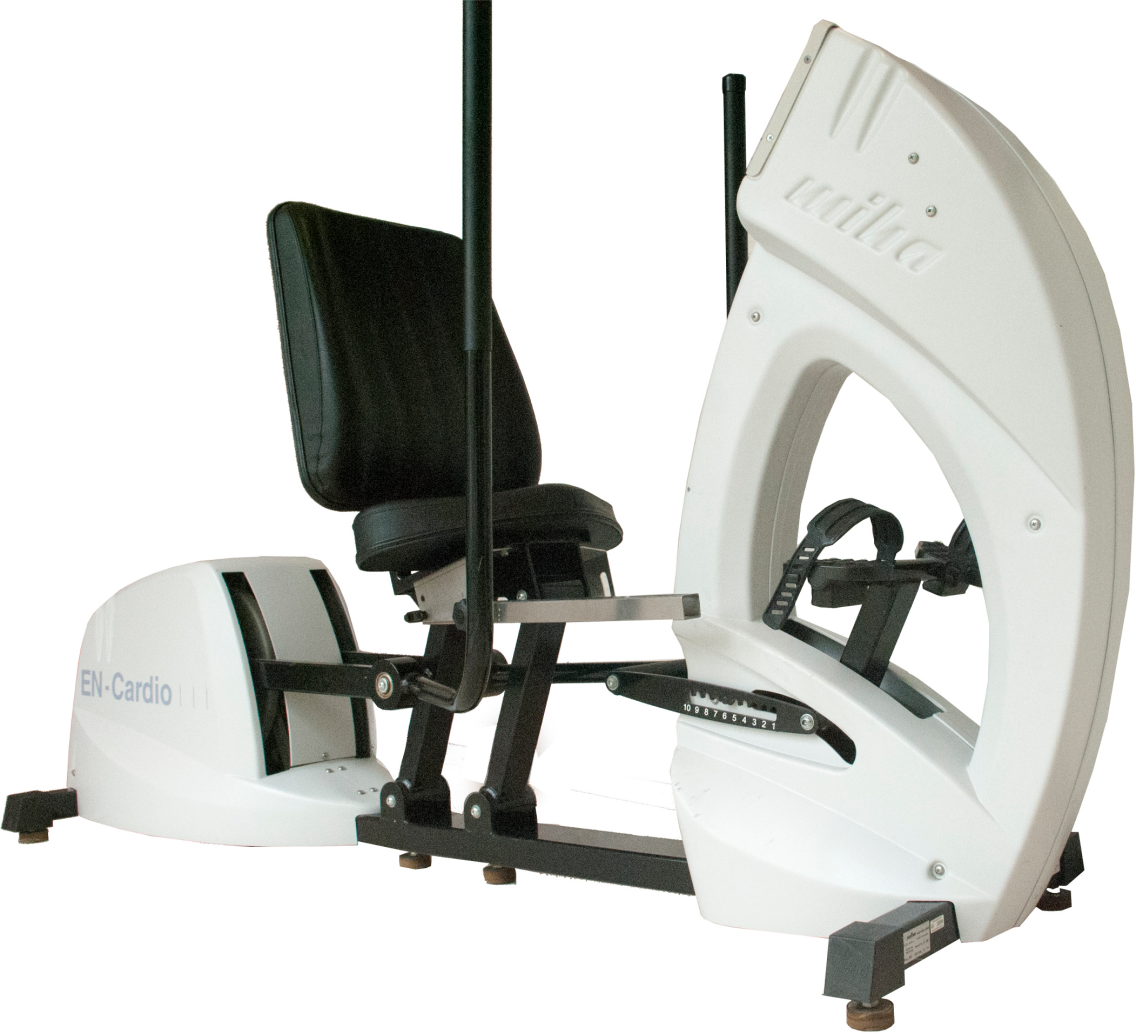
The Cruiser ergometer.

The Cruiser ergometer (Enraf-Nonius serial number: 3800EN014, ultimo number 10–3013, Delft, The Netherlands) was standardized for use in exercise testing. It is a combined arm-leg ergometer equipped with a comfortable seat (Fig 1). The foot of the user is placed against a fixed footrest, which can be adjusted to the subjects’ length. Users can perform tests on the ergometer without their prosthesis. The residual limb is supported by a special stump support connected to the seat. The footrest is used to push off and make the seat move backward. Users can move the seat forward again by pulling the handlebars. In this way, arms and leg are simultaneously used to overcome the resistance provided by the ergometer. The position of the footrest of the ergometer was adjusted to a fixed setting for each subject during the tests. The ergometer could only be set in a constant power mode of between 35 and 60 revolutions per minute (rpm) and subjects were instructed to maintain 50 rpm [12–14]. The accuracy of the Cruiser ergometer is ± 10% power output (PO in W) and ± 2 rpm speed. Cardiorespiratory parameters were recorded using an Oxycon Delta (Jaeger, Bunnik, the Netherlands). Subjects wore a face mask and ventilation (VE in l/min), oxygen uptake (VO_2_ in l/min), and carbon dioxide output (VCO_2_ in l/min) were continuously measured. Peak VO_2_ and peak VCO_2_ were defined as the highest average values obtained over a 30- s period. Blood pressure was measured manually at the beginning of the test, immediately after the test was completed, and after the cooling down period. Heart rate (HR in b/min) was continuously recorded with a 12-lead electrocardiogram (ECG).

### Study design

All subjects performed 4 exercise tests (1 submaximal and 3 maximal tests) on 3 different days, with an interval of more than 1 week between each test day. On day 1, the principal investigator screened the subjects for contraindications by means of a questionnaire, ECG, and measurement of blood pressure. Next, the subjects started the submaximal test to become acquainted with the Cruiser ergometer and to determine the gross mechanical efficiency (GE). The submaximal exercise test consisted of 3 minutes rest on the Cruiser followed by 3 minutes exercise at 20 W and 3 minutes exercise at 30 W at 50 rpm. GE of the participants was measured during the final 30 seconds of the submaximal blocks of 20 and 30 W. GE was determined to analyze the mechanical efficiency of the movement on the Cruiser ergometer. GE is an important measure because it can be used to evaluate future training effects as well as motor learning effects of the Cruiser ergometer [13,14]. After the submaximal test, subjects had a rest period of more than 10 minutes, which was followed by the first maximal exercise test. The maximal exercise test was repeated on days 2 and 3. The first maximal exercise test was seen as a familiarization test, and tests 2 and 3 were used to determine test-retest reliability. Each maximal exercise test started with 3 minutes rest on the Cruiser and was followed by a 3 minute warm-up at 20 W at 50 rpm. After the warm-up, work load was increased by 10 W per minute, keeping speed at 50 rpm, until the point of exhaustion was reached or until the physician stopped the test. Reasons for terminating the test were inability to maintain 50 rpm, pain in arms or legs, chest pain, dizziness or faintness, severe dyspnea, pallor, cyanosis, or cold and clammy skin. The test was also stopped by the investigator in case of ECG abnormalities. After completing the test, subjects were observed for another 3 minutes. The protocol was derived from an earlier study in healthy volunteers [12]. Taking the lower exercise capacity of subjects with LLA into account, appropriate adaptations in workload were made. The maximal exercise test protocol was consistent in all subjects over the 3 test days.

### Outcome

#### Feasibility

To evaluate the feasibility of the Cruiser ergometer as an instrument for measuring the physical fitness of subjects with a LLA, this study specifically focused on the indicators acceptability, demand, and practicality [15]. To this end, a 5-step approach was used. First, it was investigated whether all relevant variables could be measured during the test. Second, subjects who dropped out of the study were analyzed, and this analysis served to refine exclusion criteria for an exercise test on the Cruiser ergometer. Third, when subjects experienced symptoms of dyspnea and fatigue, these symptoms were assessed using a 10-point Borg scale at peak load for dyspnea and for arm and leg muscle fatigue so as to determine the impact of the test [16]. Fourth, the maximal aerobic performance was evaluated for each test. Since no reference data are available of the maximal aerobic performance on the Cruiser ergometer using one leg and two arms, the criteria for the bicycle ergometer were used. A performance was regarded as a maximal aerobic performance when 1 or more of 3 criteria were fulfilled: a heart rate of more than 85% of predicted, maximal ventilation of more than 75% of predicted, or a respiratory exchange rate (RER) of more than 1.1 [17]. In addition, it was studied to what extent VO_2_peak was related to predicted VO_2_peak, which is age and gender dependent (calculated for the bicycle ergometer). Fifth, the GE of movement during the submaximal period of 20 and 30 W was calculated and compared to previous research of Simmelink et. al in healthy volunteers [13,14]. GE (in %) is derived from the ratio between the mechanical power output (Po) and the metabolic power (P_met_), as shown in Eq 1 [18].

$$GE(\%)=\frac{Po}{Pmet}100\%$$ (1)

The metabolic power (Pmet) was calculated using Eq 2.

$$P_{met}(w)={VO}_{2}[\frac{\left(4.940\;RER+16040\right)}{60}]$$ (2)

#### Safety

Subjects became acquainted with the Cruiser ergometer during the submaximal exercise test. When no complications occurred, they started the maximal exercise test. It was studied whether ECG and blood pressure could be reliably recorded prior to, during, and after the (sub)maximal exercise tests. Outcome measures for safety were ECG abnormalities or the occurrence of adverse events during the tests.

#### Reliability

Test-retest reliability of the maximal exercise test on the Cruiser was studied by comparing outcome measures of the second and third tests. Outcome measures for reliability were peak oxygen uptake (VO_2_ peak), peak heart rate (HR), peak power output (PO), peak ventilation (VE), peak breathing frequency (BF), and respiratory exchange rate (RER).

### Statistics

Data analyses were performed using SPSS version 23.0 for Windows (SPSS, Chicago, Illinois, USA). Descriptive statistics were generated for all variables (mean ± SD).

Reliability of the second and third maximal exercise tests was assessed by means of paired t-tests, the one-way random effect model and single measure-intraclass correlation coefficient (ICC), and Bland-Altman plots. Level of significance was set at p <0.05.

## Results

Between May 2012 and September 2015 a total of 21 subjects were screened for inclusion. One subject was excluded because of a Syme amputation, which made it impossible to perform the tests without using a prosthesis. Three other subjects dropped out of the study due to medical reasons. Consequently, the complete test results of 17 subjects (14 men and 3 women) could be obtained. Mean age was 54.5 y (SD 18.6, range 25–80), mean BMI 25.2 kg/m^2^ (SD 4.0, range 18.4–31.2), and the mean time since amputation was 96.6 months (SD 111.2, range 2–372). Subject characteristics and outcome measures are shown in Table 1.

**Table 1:** Individual data of the submaximal and 3 maximal exercise tests on the Cruiser ergometer in 17 subjects with a lower limb amputation (mean scores with standard deviation (SD)).

Subject Characteristics							GE		1st Cruiser test							2nd Cruiser test							3rd Cruiser test						
sex	amplevel	time since amp	Reas for amp	use of B-bloc	Age (y)	BMI (kg/m2)	20W	30W	HR (b/min)	PO (W)	RER	VO_2_ (l/min)	VO_2_/ VO_2_pred (%)	BF(br/min)	VE (l/min)	HR (b/min)	PO (W)	RER	VO_2_ (l/min)	VO_2_/ VO_2_ pred(%)	BF (br/min)	VE (l/min)	HR (b/min)	PO (W)	RER	VO_2_ (l/min)	VO_2_/ VO_2_ pred (%)	BF (br/min)	VE (l/min)
M	TF	4	cancer	yes	66	27.2	10.3	8.6	142	70	1.18	1.16	49	25	50	95	50	1.00	0.94	40	24	31	148	90	1.20	1.21	52	26	50
M	TF	13	trauma	no	26	20.7		10.3	120	90	1.08	1.49	45	16	38	128	100	1.23	1.38	41	16	38	122	100	1.14	1.52	46	19	41
M	TT	2	vasdi	no	71	23.9	5.4		150	20	1.12	1.59	79	14	59	158	20	1.22	1.47	73	34	77	133	20	1.25	1.40	69	30	76
M	TT	156	trauma	no	48	28.7	8.0	6.8								139	140	1.07	2.58	104	33	83	112	100	1.02	1.69	68	28	46
M	TF	2	painsy	no	73	23.8	8.0	5.5	145	60	1.09	1.51	75	25	46	142	90	1.11	1.82	88	23	53	153	90	1.07	1.87	90	27	51
F	TT	30	trauma	no	31	18.6	14.4	12.0	165	90	1.2	1.26	69	30	42	147	70	1.12	1.14	62	24	33	171	100	1.24	1.35	56	24	47
M	TF	3	trauma	no	53	27.0	13.8	8.3	156	100	1.01	1.56	65	26	55	143	100	1.03	1.49	63	23	50	145	100	1.04	1.52	64	25	52
M	TF	24	trauma	no	67	27.1	10.6	8.8	126	40	0.95	0.93	46	27	38	96	40	0.83	0.76	36	25	27	133	30	0.92	0.84	40	32	38
M	TT	4	vasdi	no	61	18.4	15.7	9.9	150	70	1	1.12	61	26	42	139	70	1.03	0.98	54	25	42	142	80	1.11	1.14	63	26	48
M	KD	84	neufib	no	33	27.2	9.2	6.9	139	100	1	1.96	40	27	53	165	110	0.98	1.92	61	25	55	173	120	1.07	1.92	61	29	71
F	TF	132	cancer	no	25	26.1	10.0	8.0	184	130	1.1	2.29	117	47	73	186	130	1.01	1.99	102	37	59	184	130	1.08	1.87	96	34	52
F	KD	96	trauma	no	40	19.1	18.1	14.0	165	80	0.96	1.40	87	28	38	186	90	1.12	1.39	88	25	43	194	100	1.15	1.49	94	29	54
M	TF	324	trauma	no	80	28.4	2.7		143	20	1.14	2.23	130	44	80	140	20	1.22	2.02	118	50	82	134	20	1.36	1.82	106	49	70
M	TT	372	trauma	yes	78	27.8	15.0	7.9	186	60	1.04	1.31	71	20	38	126	70	0.93	1.31	70	21	43	139	70	1.00	1.31	70	21	43
M	TT	132	trauma	no	68	31.2	9.7	8.3	152	190	1.1	3.06	142	42	107	134	160	0.95	2.56	118	33	72	152	180	1.05	3.05	141	41	97
M	TT	108	trauma	no	43	23.0	13.2	10.1	117	100	1.05	1.66	59	24	48	105	110	1.01	1.76	63	20	49	122	110	1.00	1.63	58	17	41
M	TF	156	trauma	no	64	30.0	10.3	9.1	102	100	1.13	1.41	64	17	46	103	100	1.10	1.36	62	18	41	117	110	1.06	1.57	71	19	46
Mean		96.6			54.5	25.2	10.9	9.0	146.4	82.5	1.07	1.62	74.9	27.4	53.3	137.2	86.5	1.06*	1.58	73.1	28.0	51.7	145.5	91.2	1.11*	1.60	73,2	26.8	54.3
SD		111.2			18.5	4.0	4.0	2.1	22.9	41.7	0.07	0.54	30.3	9.6	18.9	27.4	39.5	0.11	0.52	25.9	8.0	17.6	23.6	40.1	0.11	0.47	25.2	8.3	15.5

Abbreviations: GE = Gross Efficiency in percentage (%); M = male, F = female; Amp level = amputation level; TF = transfemoral, TT = transtibial, KD = knee disarticulation; time since amp = time since amputation in months; Reas for amp = reason for amputation, vasdi = vascular disease, painsy = pain syndrome, neufib = neurofibromatosis; Age in years; BMI = body mass index in kg/m^2^; HR = peak heart rate in beats/minute; PO = peak power output in Watt; RER; respiratory exchange rate at the end of the test; VO_2_ = peak oxygen uptake in liter/minute; VO_2_/VO_2_pred = peak oxygen uptake in relation to the predicted oxygen uptake in percentage; BF = peak breathing frequency in breaths/minute; VE = peak ventilation in liter/minute.

* p<0.05 for paired sample test between second and third test.

### Feasibility

Three subjects dropped out of the study due to medical reasons. Subject 1 developed an allergic skin reaction located at the site of the ECG suction cups after the first day. The subject recovered within three days but did not perform days 2 and 3 of the protocol. Subject 2 showed signs of cardiac ischemia during the first maximal exercise test and was referred to a cardiologist. This subject withdrew from the study. Subject 3 dropped out after day 2 because of an increase in stump pain. Another subject had hypertension on day 1 (196/103 mmHg) and was referred to his general practitioner. This subject, however, could re-enter the study after treatment. All subjects who completed the protocol (n = 17) were able to perform the movement on the Cruiser ergometer. In most cases (second test 9/17 subjects, third test 11/17 subjects), the chosen protocol led to an exercise time of 10–15 minutes, which is considered to be the ideal duration of a maximal exercise test. Monitoring of cardiopulmonary parameters was possible in all subjects.

The Borg scores, though relatively low, were fairly consistent for all of the tests (Table 2). The Borg score for arm fatigue was significantly higher for the first maximal test than for the second test. There were no significant differences between the tests with regard to other Borg scores.

**Table 2:** Borg scores of the 3 maximal exercise tests in 17 subjects with a lower limb amputation.

	Max test 1 Mean (SD)	Max test 2 Mean (SD)	Max test 3 Mean (SD)
Dyspnea peak	2.9 (3.0)	2.9 (2.0)	3.0 (2.4)
Leg fatigue peak	3.2 (2.8)	2.8 (2.6)	3.6 (2.8)
Arm fatigue peak	3.9 (2.5) *	3.0 (2.6)	3.5 (2.1)

*significant difference between Borg score for fatigue of the arms between first and second test on the Cruiser ergometer

The majority of the subjects had a maximal aerobic performance: 11 of 16 subjects performed maximal at test 1, 12 of 17 subjects performed maximal at test 2, and 14 of 17 subjects performed maximal at test 3. VO_2_ peak was 75% of predicted (SD 30, range 40–140%) for test 1; for test 2 it was 73% of predicted (SD 26, range 36–118%); and for test 3 it was also 73% of predicted (SD 25, range 40–141%) (Table 1). In most cases, the test was stopped because coordination problems of arms and leg, which resulted in an inability to maintain the speed of 50 rpm (Table 3).

**Table 3:** Reasons for stopping the maximal exercise test.

	Test 1 n = 16	Test 2 n = 17	Test 3 n = 17
Coordination problems (%)	37.5% (6/16)	47.1% (8/17)	52.9% (9/17)
ECG abnormalities (%)	31.3% (5/16)	23.5% (4/17)	17.7% (3/17)
Muscle fatigue in the leg (%)	18.8% (3/16)	17.7% (3/17)	17.7% (3/17)
Muscle fatigue in the arms (%)	6.3% (1/16)	5.9% (1/17)	5.9% (1/17)
Dyspnea (%)	6.3% (1/16)	5.9% (1/17)	5.9% (1/17)

The mean GE at 20 W was 10.9% (SD 4.0, range 2.7–19.1, n = 16) with one missing data point and 9.0% (SD 2.1, range 5.5–14.0, n = 15) at 30 W with two missing data points (Table 1).

### Safety

Most of the tests were performed without complications. As mentioned earlier, three adverse events occurred, resulting in attrition of three subjects. These adverse events were an allergic skin reaction due to the ECG suction cups, an abnormal ECG, and an increase in pain of the stump without physical signs after exercising on the Cruiser ergometer. The ECG could be reliably recorded in all subjects, at least during low to medium workloads. The investigator had to stop a number of tests because the ECG was affected by muscle activity of the arms and thorax (Table 3). Blood pressure could not be reliably measured during exercise, but only before and after the test.

### Reliability

The first maximal exercise test was a familiarization trial. Tests 2 and 3 were used for test-retest reliability analysis. No significant differences were present, except in RER (Table 1). A slight learning/ practice effect was seen, especially with regard to the peak workload in the 3 sequential maximal exercise tests.

The ICCs are presented in Table 4. It is advocated that an ICC of > 0.75 indicates good agreement [19,20]. This means there is a good agreement for the outcome measures VO_2_peak and POpeak between tests 2 and 3.

**Table 4:** ICC of the second and third test for peak oxygen uptake, heart rate, and power output.

	ICC (single measure)	95% CI
VO_2_, l/min	0.84	0.61–0.94
HR, b/min	0.68	0.32–0.87
PO, W	0.91	0.77–0.97

Abbreviations: VO_2_ = peak oxygen uptake, HR = peak heart rate, PO = peak power output; ICC = intraclass correlation coefficient, 95% CI = 95% confidence interval.

**Fig 2:**
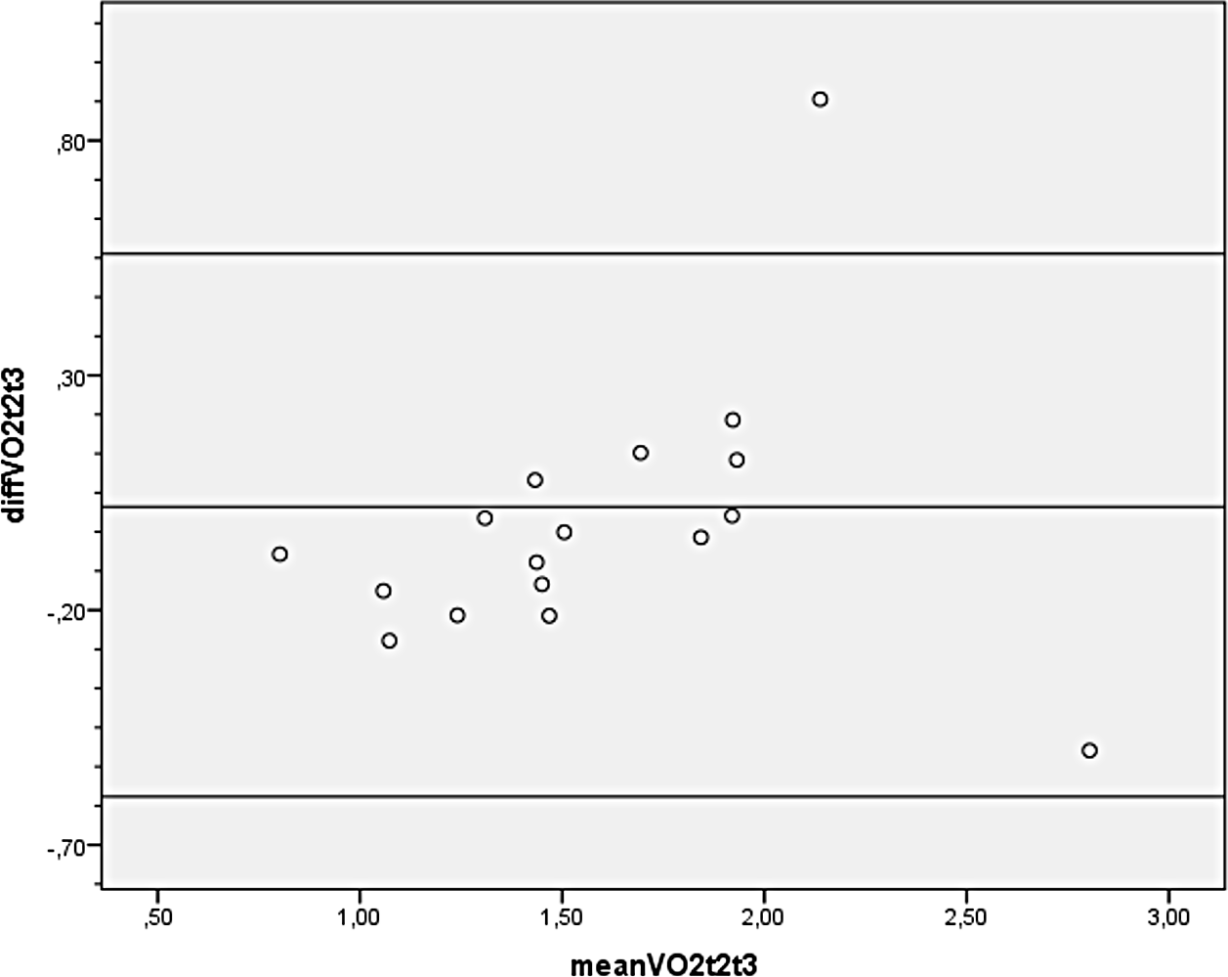
Bland-Altman plot for peak oxygen uptake of 17 subjects with a lower limb amputation during exercise testing on the Cruiser ergometer. diff VO2t2t3 = difference between peak oxygen uptake in l/min between test 2 and test 3. mean VO2t2t3 = mean peak oxygen uptake in l/min of test 2 and test 3.

**Fig 3:**
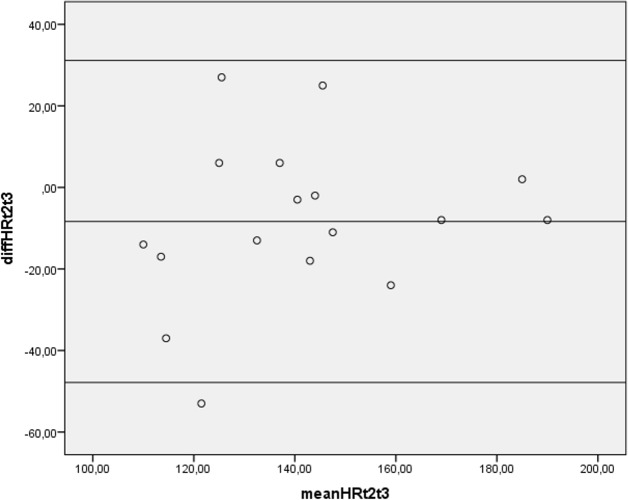
Bland-Altman plot for peak heart rate of the exercise test on the Cruiser ergometer of 17 subjects with a lower limb amputation. diffHRt2t3 = difference between peak heart rate (b/min) between test 2 and test 3 on the Cruiser ergometer. mean HRt2t3 = mean peak heart rate (b/min) of test 2 and test 3 on the Cruiser ergometer.

**Fig 4:**
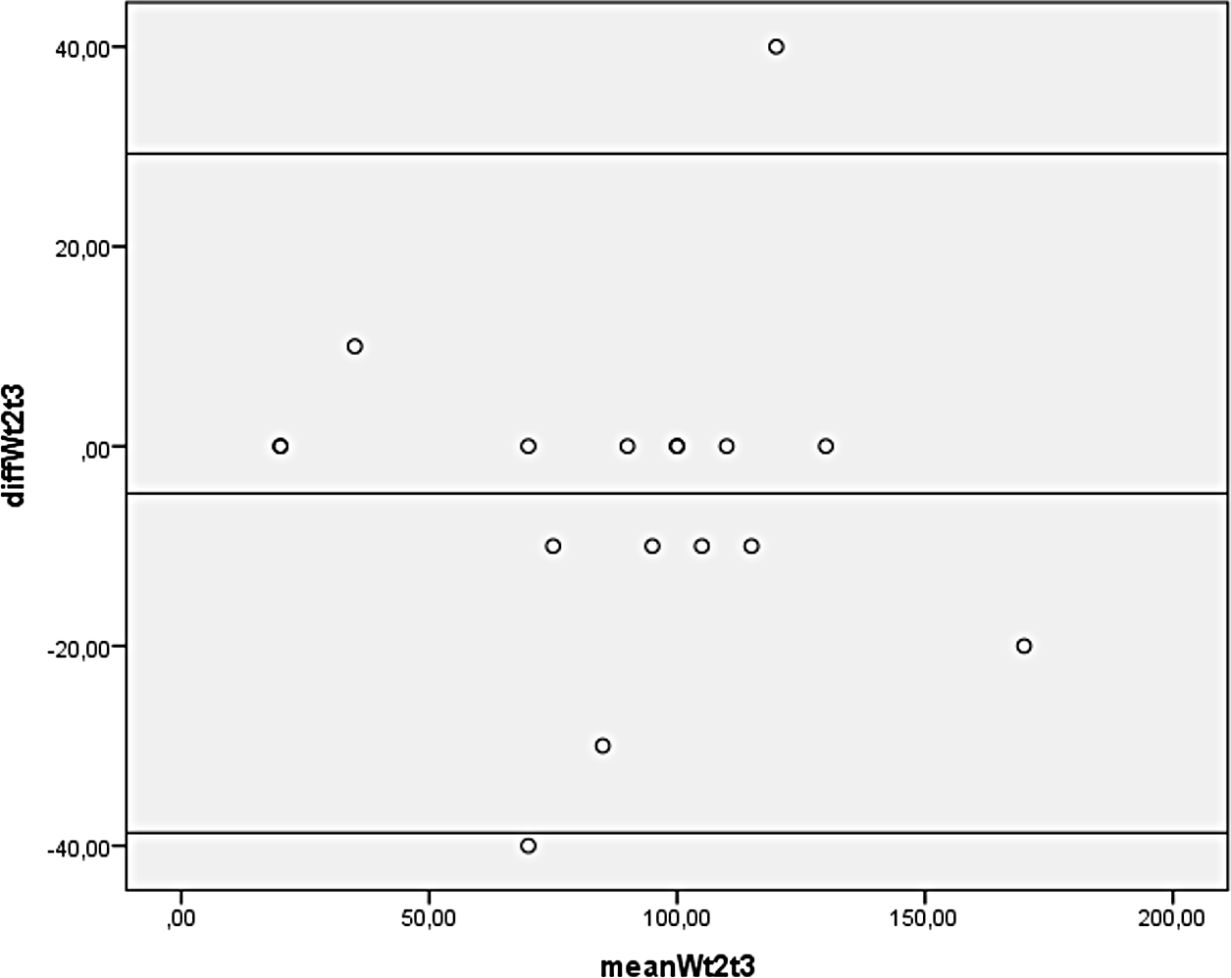
Bland-Altman plot for peak power output of the exercise test on the Cruiser ergometer of 17 subjects with a lower limb amputation. diffWt2t3 = difference between peak power output in Watt between test 2 and test 3. mean Wt2t3 = mean peak power output in Watt of test 2 and test 3.

Finally, Bland-Altman plots were constructed. The plot for VO_2_ shows a bias close to zero with two outliers (Fig 2). Limits of agreement are 0.02 ± 0.58 (mean difference ±2 SD). The plot for HR shows a similar pattern (Fig 3), although this plot has only one outlier. Limits of agreement are -8.4 ±39.5. Furthermore, the Bland-Altman plot for PO (Fig 4) has a bias close to zero with 2 outliners. Limits of agreement are -4.7±34.0.

## Discussion

In this study, feasibility, safety, and reliability of exercise testing on the combined arm-leg Cruiser ergometer were evaluated in subjects with a LLA. The test procedure was feasible because almost all subjects were able to perform the combined arm-leg movement and managed to reach adequate symptom scores. Furthermore, a large majority of subjects reached a maximal aerobic performance and most variables of interest could be measured appropriately. The test procedure was safe and ECGs could be reliably recorded in most cases. Approximately 20% of ECG recordings were severely influenced by muscle activity of arms and thorax. In addition, blood pressure could not be measured during the tests on the upper arm or on the wrist. Finally, the test-retest reliability was good.

### Feasibility

Three subjects dropped out of this study. One subject had an allergic skin reaction to the ECG suction cups. Although the skin reaction healed spontaneously within a few days, the subject was withdrawn from the study. ECGs are now in clinical practice recorded using self-adhesive electrodes. Another subject dropped out because his pre-existent stump pain worsened after test 2, despite the use of a special stump support attached to the Cruiser. Most subjects, however, felt the stump support was sufficiently comfortable and did not experience pain or discomfort in their stump.

Borg scores for dyspnea and fatigue of the arms and leg were relatively low (mean scores between 2.8 and 3.9). In a study on healthy volunteers, Borg scores for fatigue of the arms (4.9–5.0) and legs (4.3–4.5) were higher [12]. It could be hypothesized that the real maximal aerobic capacity was not measured in our study population. In the study of Wezenberg [21], 93% of subjects reached a RER peak value of more than 1.1. In our study, at least 1 of the following 3 criteria had to be fulfilled in order to consider a test as a maximal aerobic performance: a heart rate of more than 85% of predicted, maximal ventilation of more than 75% of predicted, and a RER of more than 1.1 [17]. Following these criteria, at the first test 69% (n = 11/16) of subjects had a maximal exercise test and at the second and thirds test 71% (n = 12/17) and 82% (n = 14/17) of subjects had a maximal exercise test, respectively. These scores may be explained by several factors. Subjects may have experienced increased symptom perception due to the unfamiliar type of exercise, to coordination problems, and to the relatively large impact of arm exercise in relation to leg exercise. In addition, a few tests were discontinued due to ECG disruptions. Finally, the fixed protocol with relatively large increments of workload may have been less appropriate.

Some learning effects were found, including higher VO2 and greater power output, which have also been demonstrated in previous research [12]. The GE varied greatly between subjects and ranged between 2.7–18.1% at 20 W and 5.5–14.0%. at 30 W. In earlier research in healthy young subjects, a GE of 13.0 in men and 15.0% in women at 45 W was found [13]. When using the Cruiser ergometer in the future for testing and training, it is important to realize that motor learning effects can vary among subjects.

### Safety

Although ECG recordings were possible during the exercise test on the Cruiser, they were hampered by muscle activity of arms and thorax. Nevertheless, ECG abnormalities were found in one subject, who was referred to a cardiologist. Exercise stress testing on the bicycle ergometer has a sensitivity of 33–50% and a specificity of 84–96% for detecting coronary artery disease in patients with suspected myocardial ischemia. ECG abnormalities are more easily detected after stopping the exercise. Therefore, in clinical practice, ECGs are recorded 2 minutes after the exercise phase [22,23]. In addition, blood pressure could not be measured during the test, neither with an upper arm monitor or manually measured nor with a pulse monitor This is a limitation of the Cruiser test with respect to safety especially for patients with LLA with a high risk of cardiovascular disease. In this study, however, no subject had a systolic blood pressure of > 225 mm Hg or a diastolic blood pressure of > 99 mmHg directly after the exercise phase. In future research measurement of blood pressure may be possible, e.g. by using a discontinuous protocol. In addition, further research into the question whether blood pressure measurement before and directly after the test is sufficient, has to be performed. Finally, with the exception of the three subjects who dropped out of the study due to medical reasons, no other subjects experienced medical problems during or after the tests.

### Reliability

Reliability was measured by comparing the second and third test on the Cruiser ergometer and was found to be satisfactory in general, as can be deducted from the ICC and Bland-Altman plots. However, the Bland-Altman plots revealed some outliers. The outliers may be explained by a difference in effort of some subjects between the second and third test and by prematurely stopping the test in 7 subjects because of suspected ECG abnormalities. In retrospect, the premature stop of the tests was not necessary, for the abnormalities could be explained by muscle activity in all but one subject. It is recommended that both subject and investigator learn how to use the Cruiser ergometer prior to testing so as to improve reliability.

### Limitations of this study

As shown in Table 1, physical fitness of the study population varied greatly. This is to be expected in patients with LLA, who follow a rehabilitation program to learn to walk with a prosthesis. Most subjects in our study were trauma patients, which is unusual given that peripheral arterial disease is the main reason for performing a LLA [24]. It is likely that selection bias occurred in this study. Specialists in Rehabilitation Medicine and certified prosthetists located in the North of the Netherlands were asked by the principal investigator to recruit subjects with a LLA for this study. This might have led to a selection bias of motivated and relatively healthy subjects. Consequently, the findings of this study might overestimate the physical fitness of this population The study population was not large enough to perform a comparative analysis between amputees with different causes of the amputation such as trauma, vascular disease, cancer, pain syndrome and neurofibromatosis. The time since amputation varied between 2 to 372 months, which is a large variation. This variation, however, enabled us to study the Cruiser in both inexperienced and experienced subjects. No differences were seen with regard to ease of use and becoming acquainted with the Cruiser ergometer. One protocol was used in all subjects. For some subjects, the submaximal level of 30 W was already the maximal power output they could reach on the Cruiser ergometer. Two subjects only reached a peak power output of 20 W, which was still a maximal aerobic performance when considering the criteria for maximal aerobic exercise. In future studies, individually tailored exercise protocols are needed instead of a one-size-fits-all protocol. These protocols should aim at a test duration of 10–15 minutes [19].

### Recommendations for further research

An implementation study is recommended to design patient- specific protocols for using the Cruiser ergometer at the start of the rehabilitation period of patients with a LLA. Also, future research is needed to design exercise training protocols using the data from the baseline Cruiser exercise test.

## Conclusions

The Cruiser ergometer is a feasible, safe, and reliable testing instrument for measuring the physical fitness of this study population with LLA. Adequate practice before actual testing, and the development of individually tailored testing protocols are advised.

## Supporting information

S1 Dataset (PDF)

## References

[1] Remes L, Isoaho R, Vahlberg T, Hiekkanen H, Korhonen K, Viitanen M, et al Major lower extremity amputation in elderly patients with peripheral arterial disease: incidence and survival rates. Aging Clin Exp Res. 2008;20: 385–393. 19039278 10.1007/BF03325142

[2] Kaptein S, Geertzen JHB, Dijkstra PU. Association between cardiovascular diseases and mobility in persons with lower limb amputation: a systematic review. Disabil Rehabil. 2018;40: 883–888. 10.1080/09638288.2016.1277401 28129515

[3] Kahle JT, Highsmith MJ, Schaepper H, Johannesson A, Orendurff MS, Kaufman K. Predicting Walking Ability Following Lower Limb Amputation: an Updated Systematic Literature Review. Technol Innov. 2016;18: 125–137. 10.21300/18.2-3.2016.125 28066522 PMC5218540

[4] Chin T, Sawamura S, Fujita H, Ojima I, Oyabu H, Nagakura Y, et al %VO2max as an indicator of prosthetic rehabilitation outcome after dysvascular amputation. Prosthet Orthot Int. 2002;26: 44–49. 10.1080/03093640208726620 12043925

[5] Wezenberg D, van der Woude L H, Faber WX, de Haan A, Houdijk H. Relation between aerobic capacity and walking ability in older adults with a lower-limb amputation. Arch Phys Med Rehabil. 2013;94: 1714–1720. 10.1016/j.apmr.2013.02.016 23466292

[6] van Velzen JM, van Bennekom CA, Polomski W, Slootman JR, van der Woude L H, Houdijk H. Physical capacity and walking ability after lower limb amputation: a systematic review. Clin Rehabil. 2006;20: 999–1016. 10.1177/0269215506070700 17065543

[7] Chin T, Sawamura S, Shiba R. Effect of physical fitness on prosthetic ambulation in elderly amputees. Am J Phys Med Rehabil. 2006;85: 992–996. 10.1097/01.phm.0000247653.11780.0b 17117003

[8] Riebe D, Franklin BA, Thompson PD, Garber CE, Whitfield GP, Magal M, et al Updating ACSM's Recommendations for Exercise Preparticipation Health Screening. Med Sci Sports Exerc. 2015;47: 2473–2479. 10.1249/MSS.0000000000000664 26473759

[9] Bostom AG, Bates E, Mazzarella N, Block E, Adler J. Ergometer modification for combined arm-leg use by lower extremity amputees in cardiovascular testing and training. Arch Phys Med Rehabil. 1987;68: 244–247. 3566520

[10] Erjavec T, Vidmar G, Burger H. Exercise testing as a screening measure for ability to walk with aprosthesis after transfemoral amputation due to peripheral vascular disease. Disabil Rehabil. 2014;36: 1148–1155. 10.3109/09638288.2013.833307 24020425

[11] Vestering MM, Schoppen T, Dekker R, Wempe J, Geertzen JH. Development of an exercise testing protocol for patients with a lower limb amputation: results of a pilot study. Int J Rehabil Res. 2005;28: 237–244. 16046917 10.1097/00004356-200509000-00006

[12] Simmelink EK, Wempe JB, Geertzen JH, Dekker R. Repeatability and validity of the combined arm-leg (Cruiser) ergometer. Int J Rehabil Res. 2009;32: 324–330. 10.1097/MRR.0b013e328325a8a8 19252438

[13] Simmelink EK, Borgesius EC, Hettinga FJ, Geertzen JH, Dekker R, van der Woude L H. Gross mechanical efficiency of the combined arm-leg (Cruiser) ergometer: a comparison with the bicycle ergometer and handbike. Int J Rehabil Res. 2015;38: 61–67. 10.1097/MRR.0000000000000100 25419689

[14] Simmelink EK, Wervelman T, de Vries HS, Geertzen JHB, Dekker R, van der Woude L H V. One-day low-intensity combined arm-leg (Cruiser) ergometer exercise intervention: cardiorespiratory strain and gross mechanical efficiency in one-legged and two-legged exercise. Int J Rehabil Res. 2017;40: 347–352. 10.1097/MRR.0000000000000251 28877043

[15] Bowen DJ, Kreuter M, Spring B, Cofta-Woerpel L, Linnan L, Weiner D, et al How we design feasibility studies. Am J Prev Med. 2009;36: 452–457. 10.1016/j.amepre.2009.02.002 19362699 PMC2859314

[16] Borg GA. Psychophysical bases of perceived exertion. Med Sci Sports Exerc. 1982;14: 377–381. 7154893

[17] Rieger BW. Principles of Exercise Testing and Interpretation: Including Pathophysiology and Clinical Applications, 3rd Edition. Medicine & Science in Sports & Exercise. 2000;32: 1364.

[18] Garby L, Astrup A. The relationship between the respiratory quotient and the energy equivalent of oxygen during simultaneous glucose and lipid oxidation and lipogenesis. Acta Physiol Scand. 1987;129: 443–444. 3577829 10.1111/j.1365-201x.1987.tb10613.x

[19] Lee J, Koh D, Ong CN. Statistical evaluation of agreement between two methods for measuring a quantitative variable. Comput Biol Med. 1989;19: 61–70. 2917462 10.1016/0010-4825(89)90036-x

[20] Tammemagi MC, Frank JW, Leblanc M, Artsob H, Streiner DL. Methodological issues in assessing reproducibility—a comparative study of various indices of reproducibility applied to repeat ELISA serologic tests for Lyme disease. J Clin Epidemiol. 1995;48: 1123–1132. 7636514 10.1016/0895-4356(94)00243-j

[21] Wezenberg D, de Haan A, van der Woude L H, Houdijk H. Feasibility and validity of a graded one-legged cycle exercise test to determine peak aerobic capacity in older people with a lower-limb amputation. Phys Ther. 2012;92: 329–338. 10.2522/ptj.20110125 22156028

[22] Puelacher C, Wagener M, Abacherli R, Honegger U, Lhasam N, Schaerli N, et al Diagnostic value of ST-segment deviations during cardiac exercise stress testing: Systematic comparison of different ECG leads and time-points. Int J Cardiol. 2017;238: 166–172. 10.1016/j.ijcard.2017.02.079 28320607

[23] Task Force Members, Montalescot G, Sechtem U, Achenbach S, Andreotti F, Arden C, et al 2013 ESC guidelines on the management of stable coronary artery disease: the Task Force on the management of stable coronary artery disease of the European Society of Cardiology. Eur Heart J. 2013;34: 2949–3003. 10.1093/eurheartj/eht296 23996286

[24] Fortington LV, Rommers GM, Postema K, van Netten JJ, Geertzen JH, Dijkstra PU. Lower limb amputation in Northern Netherlands: unchanged incidence from 1991–1992 to 2003–2004. Prosthet Orthot Int. 2013;37: 305–310. 10.1177/0309364612469385 23327835

