# Supplementary material for: Feasibility, safety, and reliability of exercise testing using the combined arm-leg (Cruiser) ergometer in subjects with a lower limb amputation
Source: PLoS One. 2018 Aug 13;13(8):e0202264. doi: 10.1371/journal.pone.0202264 (PMC6089442; doi:10.1371/journal.pone.0202264)
Supplement: S1 Dataset — (PDF) [file pone.0202264.s001.pdf]

	geslacht	amplevel	time after a	side amput	cause amp	B-blockers	age	length (cm)
P 1	man	transfem	4	right	oncology	yes	66	189
P2	man	transfem	13	left	trauma	no	26	189
P4	man	transtib	2	left	vasc disea:	no	71	184
P5	man	transtib	156	right	trauma	no	48	170
P6	man	transfem	2	left	pain syndro	no	73	190
P7	woman	transtib	30	left	trauma	no	31	172
P9	man	transfem	3	right	trauma	no	53	172
P10	man	transfem	24	left	trauma	no	67	178
P11	man	transtib	4	left	vasc disea:	no	61	173
P13	man	knee-ex	84	right	neurofibror	no	33	179
P14	woman	transfem	132	right	oncology	no	25	159
P15	woman	knee-ex	96	right	trauma	no	40	162
P17	man	transfem	324	left	trauma	no	80	177
P18	man	transtib	372	left	trauma	yes	78	182
P19	man	transtib	132	left	trauma	no	68	179
P20	man	transtib	108	left	trauma	no	43	183
P21	man	transfem	156	left	trauma	no	64	177

weight(kg)	BMI (kg/m2	VO2 rest (r	VO2 sub (r	HR sub (1/	VCO2 sub	VE sub (l/n	BF sub (1/r	RER sub
97	27,2	313	981	135	1050	36	27	1,07
74	20,7	403	851	70	784	22	17	0,92
81	23,9	401	1588	150	1771	59	14	1,12
83	28,7	291	1233	92	1375	40	30	1,11
86	23,8	430	1540	127	1621	39	21	1,07
55	18,6	411	723	105	700	19	22	0,97
80	27	437	1048	124	982	34	27	0,94
86	27,1	342	970	121	986	43	33	1,02
55	18,4	353	875	127	835	31	21	0,96
87	27,2	576	1247	101	1089	31	14	0,97
66	26,1	288	1115	125	869	26	28	0,85
50	19,1	567	655	100	472	15	18	0,73
89	28,4	464	2095	145	2237	80	45	1,07
92	27,8	275	1094	148	1018	29	16	0,99
100	31,2	427	1051	65	596	24	25	0,95
77	23	296	886	97	810	22	13	0,84
94	30	293	917	72	925	31,8	19,4	1,13

GE 20 Wat	GE 30 Wat	PO sub (wa	RR syst su	RR diast su	Borg A sub	Borg B sub	Borg K sub	VO2 t1 (ml/
10,34	8,6	30	137	85				1164
	10,28	30	116	64				1490
5,25		20	120	75	5	5	7	1588
8,01	6,78	30	105	89				
7,96	5,48	30	210	85	0	0	0	1507
14,42	11,95	30	125	84	0,5	0,5	0	1254
13,83	8,3	30	127	74	0	0	0	1545
10,58	8,8	30	163	70	0,5	2	7	934
15,71	9,9	30	182	89	2	5	5	1120
9,21	6,93	30	135	85	0	0	0	1960
9,97	7,98	30	140	90	0,5	0,5	1	2291
18,13	13,99	30	130	80	0	0	0	1379
2,69		20	210	100	5	5	5	2234
14,95	7,86	30	160	95	1	0	2	1310
9,73	8,26	30	195	105	0,5	0	0	3058
13,2	10,06	30	172	68	0	2	0	1657
10,31	9,08	30	183	82	3	3	2	1410

HR t1(1/mi	VCO2 t1 (ı	VE t1 (l/mir	BF t1 (1/m	RER t1	PO t1 (watt	RR syst t1	RR diast t1	Borg A t1
142	1373	50	25	1,18	70	161	99	3
120	1586	38	16	1,08	90	127	65	8
150	1771	59	14	1,12	20	120	75	5
145	1645	46	25	1,09	60	200	75	0,5
165	1465	42	30	1,2	90	137	85	3
156	1558	55	26	1,01	100	136	78	1
126	886	38	27	0,95	40	151	62	0,5
150	1042	42	26	1	70	170	91	7
139	1757	53	27	1	100	124	76	7
184	2082	73	47	1,1	130	165	80	7
165	1290	38	28	0,96	80	145	80	2
143	2237	80	44	1,14	20	220	100	5
186	1242	38	20	1,04	60	165	90	1
152	3356	107	42	1,1	190	230	90	4
117	1813	48	24	1,05	100	157	74	4
102	1553	46	17	1,13	100	163	82	5

Borg B t1	Borg K t1	VO2 t2 (ml	HR t2 (1/m	VCO2 t2 (r	VE t2 (l/mir	BF t2 (1/mi	RER t2	POt2 (watt)
0	0	941	95	941	31	24	1	50
8	5	1378	128	1691	38	16	1,23	100
5	7	1472	158	1789	77	34	1,22	20
		2582	139	2570	83	33	1,07	140
0,5	0	1821	142	2020	53	23	1,11	90
3	3	1136	147	1272	33	24	1,12	70
1	0	1488	143	1539	50	23	1,03	100
0	9	762	96	630	27	25	0,83	40
7	5	979	139	1006	42	25	1,03	70
0	0	1921	165	1888	55	25	0,98	110
8	6	1992	186	1996	59	37	1,01	130
2	0,5	1388	186	1405	43	25	1,12	90
5	5	2025	140	2466	82	50	1,22	20
1	2	1307	126	1270	43	21	0,93	70
3	0,5	2555	134	2397	72	33	0,95	160
5	0	1762	105	1891	49	20	1,01	110
3	4	1362	103	1464	41	18	1,1	100

RR syst t2	RR diast t2	Borg A t2	Borg B t2	Borg K t2	VO2 t3 (ml	HR t3 (1/m	VCO2 t3 (n	VE t3 (l/mir
140	80	0	0	2	1206	148	1436	50
136	68	8	8	7	1523	122	1730	41
163	92	5	5	6	1395	133	1750	76
171	94	3	3	1	1694	112	1578	46
161	91	0	0	3	1866	153	1934	51
130	80	3	3	0	1347	171	1667	47
130	78	0,5	0,5	0	1522	145	1590	52
163	77	0	0	3	843	133	770	38
195	75	7	7	5	1138	142	1259	48
145	80	7	6	4	1920	173	2115	71
140	70	4	5	4	1872	184	1845	52
140	50	1	0,5	0,5	1486	194	1602	54
160	90	4	2	4	1820	134	2233	70
150	99	2	3	3	1311	139	1188	43
225	80	0,5	0	3	3054	152	3192	97
158	67	3	2	1	1627	122	1848	41
185	85	3	3	3	1574	117	1684	46

BF t3 (1/mi	RER t3	PO t3 (watt	RR syst t3	RR diast t3	Borg A t3	Borg B t3	Borg K t3	Test 1 max
26	1,2	90	141	82	1	1	3	yes
19	1,14	100	139	73	6	8	4	no
30	1,25	20	185	85	5	5	4	yes
28	1,02	100	165	97	3	3	3	
27	1,07	90	150	70	0	0	2	yes
24	1,24	100	133	88	5	5	0	yes
25	1,04	100	145	74	0,5	0,5	0	yes
32	0,92	30	149	60	1	5	5	no
26	1,11	80	199	109	7	7	8	yes
29	1,07	120	145	80	5	5	3	no
34	1,08	130	145	75	5	9	8	no
29	1,15	100	140	80	5	0,5	0,5	yes
49	1,36	20	165	90	4	4	0	yes
21	1	70	150	85	2	1	1	yes
41	1,05	180	225	95	2	0,5	3	yes
17	1	110	116	78	5	4	3	no
19	1,06	110	190	95	3	3	3	yes

Test 2 max	Test 3 max	VO2/VO2p	VO2/VO2p	VO2/VO2pred T3 (%)
no	yes	49	40	52
yes	yes	45	41	46
yes	yes	79	73	69
no	no		104	68
yes	yes	75	88	90
yes	yes	69	62	56
yes	yes	65	63	64
no	yes	46	36	40
yes	yes	61	54	63
no	yes	40	61	61
yes	yes	117	102	96
yes	yes	87	88	94
yes	yes	130	118	106
yes	yes	71	70	70
yes	yes	142	118	141
no	no	59	63	58
yes	no	64	62	71
